# Subjective and Objective Sleep Measures in Patients With Insomnia With and Without Depression

**DOI:** 10.1002/brb3.71342

**Published:** 2026-04-12

**Authors:** Tina Carbonetti, Michal Bechny, Corrado Garbazza, Jana Koprivova, Karolina Janku, Helen Christina Slawik, Annette Beatrix Bruehl, Undine Emmi Lang, Jan Sarlon

**Affiliations:** ^1^ University of Basel Basel Switzerland; ^2^ University Psychiatric Clinics Basel Basel Switzerland; ^3^ Center for Digital Health Interventions ETH Zurich Zurich Switzerland; ^4^ Centre for Chronobiology University of Basel Basel Switzerland; ^5^ National Institute of Mental Health Klecany Czech Republic; ^6^ Third Faculty of Medicine Charles University Prague Czech Republic

**Keywords:** depressive disorder, insomnia, sleep perception, subjective–objective mismatch

## Abstract

**Introduction:**

Objectively measured sleep duration often diverges from the subjectively perceived sleep duration in patients with insomnia. Although depression is known to affect sleep, little is known about its influence on the discrepancy between subjective and objective sleep measures, which we sought to clarify in this study.

**Methods:**

We analyzed medical records from 229 patients with insomnia, including anamnestic data, Pittsburgh Sleep Quality Index (PSQI) and Beck Depression Inventory (BDI) scores, and polysomnography and actigraphy measurements. Patients were compared according to their anamnesis of depressive disorder and BDI scores. Group differences were evaluated using Wilcoxon rank‐sum tests and multivariate linear regression with log‐transformed outcomes to control for relevant confounders.

**Results:**

Patients with insomnia (mean PSQI score 12.4) and comorbid depression (as a trait) showed significantly higher sleep‐onset latency, lower sleep efficiency, and longer total sleep time, as assessed by actimetry. Patients with insomnia and relevant depressive symptoms (BDI score ≥14 points) did not differ from those without relevant depression in objective sleep measurements. However, they presented significantly higher scores on subjective measurements of overall sleep quality, sleep latency, sleep disturbances, and daytime disturbances.

**Conclusions:**

In patients with insomnia, current depressive symptoms have an impact only on the subjective perception of sleep, whereas comorbid depressive disorder also influences objective sleep measurements. A clearer understanding of the discrepancy between subjective and objective sleep measures across different patient cohorts will enhance personalized treatment in patients with insomnia.

## Graphical abstract

While current depressive symptoms are associated primary with subjective sleep quality parameters, depressive disorder as a trait is linked to objective changes of the sleep architecture. All measures controlled for confounders (age, sex, comorbidities, medication).

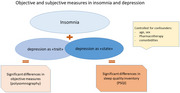

**Abbreviations**
BDI: Beck Depression Inventory
BMI: body mass index
ESS: epworth sleepiness scale
ICD: International Classification of Diseases
MDD: major depressive disorder
N1: non‐rapid eye movement sleep stage 1
N3: non‐rapid eye movement sleep stage 3 (deep sleep/slow wave sleep)
OSA: obstructive sleep apnea
PLMD: periodic limb movement disorder
PSG: polysomnography
PSQI: Pittsburgh Sleep Quality Index
REM: rapid eye movement sleep
RLS: restless legs syndrome
TST: total sleep time
UPK: University Psychiatric Clinics (Universitäre Psychiatrische Kliniken)

## 1 Introduction

Insomnia is among the most prevalent sleep disorders, affecting approximately 10% of the general population, with nearly one‐third reporting its symptoms (Morin et al. 2006). These symptoms typically involve subjective complaints of nonrestorative sleep, difficulties in initiating or maintaining sleep, or waking up earlier than desired, all of which significantly impair daytime functioning and quality of life (American Academy of Medicine 2008; Sateia 2014). Insomnia is a disabling sleep disorder that substantially impairs quality of life and increases both physical and mental health risks. It commonly co‐occurs with other mental health conditions (Sarsour et al. 2010), and evidence also indicates that chronic insomnia is an independent risk factor for the development of psychiatric disorders, including depressive disorders (Fang et al. 2019; Hertenstein et al. 2023), anxiety disorders, schizophrenia, and substance use disorders (Hertenstein et al. 2019; Palagini et al. 2022).

Previous research has consistently shown a discrepancy between subjective and objective measures of sleep quality and duration in insomnia. On average, patients with insomnia report shorter sleep duration on the Pittsburgh Sleep Quality Index (PSQI) than is recorded as total sleep time (TST) in polysomnography (PSG). Patients with insomnia underestimate their TST and overestimate their sleep latency (Harvey and Tang 2012; Maltezos et al. 2024; Valko et al. 2021).

The perception of subjective sleep parameters is more accurate for the PSG night than for long‐term actimetry measurements (Maltezos et al. 2024). In contrast, good sleeper controls tend to overestimate their actual sleep duration (Benz et al. 2023). Self‐reported, but not objective, sleep characteristics differ between individuals with current depression or anxiety and those without such conditions; patients with current depression/anxiety report both shorter and longer sleep durations and more insomnia symptoms (Difrancesco et al. 2019). Both shorter and longer sleep durations are associated with an increased risk of depression (Li et al. 2023; Zhai et al. 2015).

Although objective sleep measurements (such as PSG and actigraphy) are validated and are relevant parts of the diagnostic process (Baandrup and Jennum 2015; Moon et al. 2021), studies examining sleep duration and depression have mostly relied on self‐reported measures.

Depression is often accompanied by insomnia with less deep sleep, more rapid eye movement (REM) sleep, and shorter REM sleep latency (Riemann et al. 2020). Chronic insomnia is a known risk factor for depression (see above), and sleep disturbances can represent either a core symptom or an associated feature of depression (Fang et al. 2019; Riemann 2007; Yasugaki et al. 2025).

Although the psychophysiological mechanisms underlying the close bidirectional relationship between insomnia and depression remain unclear, there are similarities between the two disorders in terms of sleep alterations. Both disorders show signs of hyperarousal (Riemann et al. 2020), which may interfere with normal sleep. Furthermore, similar to insomnia, objective sleep parameters do not always correspond to the subjective complaints of patients with depression. Various terms can be found in the literature for describing the discrepancy between objectively recorded and subjectively perceived measures of sleep, including TST, sleep‐onset latency and wake time after sleep onset. These include “misperception index” (Castelnovo et al. 2021), “sleep state misperception” (Maltezos et al. 2024), “subjective–objective sleep discrepancy” (Stephan and Siclari 2023), and “subjective–objective mismatch” (Valko et al. 2021).

Subjective–objective sleep discrepancy has been reported in patients with major (Perlis et al. 2006) and moderate depressive disorders (Matousek et al. 2004) and has been associated with depression severity. The more severe the depression, the higher is the degree of discrepancy (Tsuchiyama et al. 2003). This subjective and objective sleep discrepancy, or sleep misperception, is thought to reflect subtle objective sleep changes not captured by conventional PSG (Parrino et al. 2012). These alterations are thought to be related to a state of hyperarousal, characterized by enhanced cortical activity and increased arousal during sleep (Spiegelhalder et al. 2012), contributing to the perception of wakefulness rather than sleep. Exploring this phenomenon is clinically important because patients with sleep misperception may be at risk of developing more severe objective sleep impairment over time (Harvey and Tang 2012). Additionally, emerging evidence suggests that sleep misperception is not merely a matter of inaccurate perception but likely reflects underlying physiological alterations in sleep that are not detected by traditional sleep measures (Rezaie et al. 2018). Gaining insights into the psychological and neurophysiological mechanisms underlying this sleep discrepancy could significantly enhance our understanding of insomnia disorders and other mental health conditions that are frequently accompanied by sleep disturbances (Dolsen et al. 2014).

In patients with sleep disorders such as insomnia, individual cognitive hyperarousal levels may be involved in negative subjective sleep perception (Zhuang et al. 2022). Some authors have suggested that this misperception may be caused by microstructural changes that cannot be detected using standard clinical PSG (Stephan and Siclari 2023). However, the findings are inconsistent; sleep fragmentation (such as non‐rapid eye movement sleep stage 1 [N1] sleep, arousal index, and sleep efficiency) is not correlated with subjective sleep duration in patients with insomnia or sleep apnea (Bianchi et al. 2013).

Castelnovo et al. (2021) investigated the relationship between psychopathology and subjective–objective sleep misperception but did not find any significant differences in misperception based on psychopathology in patients with insomnia.

This study examined how depression influences the mismatch between subjective and objective sleep measures. We compared these measures in patients with insomnia with and without depression, measured either as a trait (comorbidity of depression according to the referral) or state (Beck Depression Inventory [BDI] total score above the cutoff for major depression). Our definition of depression therefore included both current depression and a previously diagnosed depressive disorder. We decided to use the term subjective–objective mismatch, as it is more descriptive and neutral than “perception.” Consistent with previous definitions, we quantified this mismatch as the ratio of subjective to objective values, with values below 1 indicating underestimation and values above 1 indicating overestimation (e.g., 1.05 represents a 5% overestimation).

## 2 Methods

### 2.1 Study Design

In this single‐center, retrospective observational study, we analyzed data collected in routine clinical practice at the University Psychiatric Clinics (UPK) sleep clinic (see endpoints) from February 26, 2021, to April 1, 2024.

### 2.2 Primary Endpoints

The primary endpoint was the influence of depression on subjective–objective mismatch in patients with insomnia, examined in groups with and without depression and assessed separately for depression as a trait and as a state. Trait depression was defined according to the referral diagnosis, including both previous and current depression, whereas the nondepressed group included those with no history of depression. State depression was defined as a BDI total score of ≥14 versus <14 points.

The subjective–objective mismatch in sleep duration and sleep latency was defined using objective sleep measures, such as actigraphy and PSG, and subjective measures, such as the PSQI and self‐rated PSG sleep form.

### 2.3 Secondary Endpoints

Secondary endpoints included the association of depressive comorbidities with other sleep metrics, such as objective PSG‐ or actigraphy‐based measures and subjective self‐reported sleep assessments, as well as with validated scales such as the PSQI and epworth sleepiness scale (ESS).

Insomnia, as well as restless legs syndrome (RLS), obstructive sleep apnea (OSA), circadian rhythm disorders, parasomnia, and periodic limb movement disorder (PLMD), was diagnosed according to the International Classification of Sleep Disorders (American Academy of Sleep Medicine 2008). A history of depression was based on the referral diagnosis provided by the attending physician.

### 2.4 Inclusion Criteria

Patients aged 18 years and older at the UPK sleep clinic, who provided written informed consent to use healthcare‐related data recorded in the clinical routine for later analysis, were considered for the study (Appendix 1).

### 2.5 Exclusion Criteria

Patients were excluded if they did not provide written informed consent to use healthcare‐related data from the UPK; lacked subjective or objective measures of sleep duration; did not meet diagnostic criteria for insomnia; or had severe obstructive sleep apnea (defined as a respiratory disturbance index of 30 or more events per hour or any circadian rhythm disorder as classified in the International Classification of Sleep Disorders, second edition).

**FIGURE 1:**
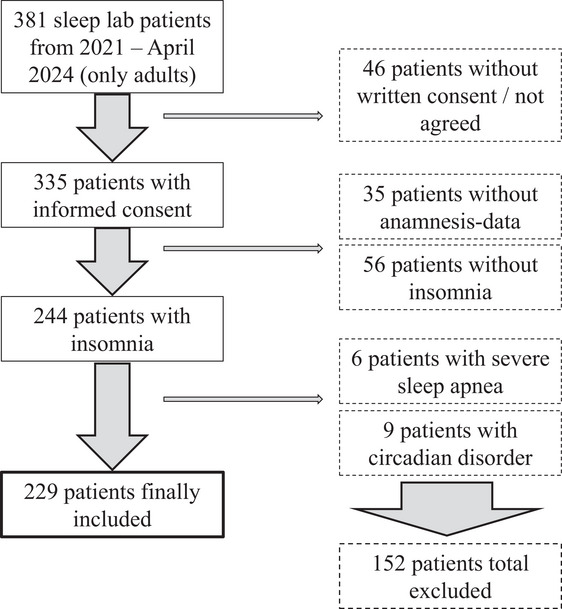
Flow‐chart depicting data selection according to exclusion criteria.

Data from 381 patients referred to the sleep laboratory of UPK Basel between 2021 and April 2024 were screened. Of these, 46 lacked informed consent or declined participation, 35 had incomplete sleep anamnesis data, 56 did not meet the diagnostic criteria for insomnia, 6 had severe obstructive sleep apnea, and 9 had a circadian rhythm disorder and were excluded. The final sample included 229 patients (Figure 1).

### 2.6 Sleep Clinic Assessment Protocol

The university sleep medicine center is reaccredited annually and consists of three main departments: pneumology, psychiatry, and neuropediatrics, headed by European Sleep Research Society (ESRS)‐certified somnologists. Although all three units can diagnose a broad spectrum of sleep disorders owing to their specialization, patients with psychiatric comorbidities are mostly referred to the psychiatric sleep clinic, and patients suffering from primary pulmonary disease are mainly referred to pneumology. Anamnestic data, including medical history and specific sleep anamnesis, were obtained from all patients. Psychotropic drug intake was defined as the intake of one or more of the following psychopharmaceuticals: hypnotics/tranquilizers (benzodiazepines and z‐substances), antidepressants, antipsychotics, and psychostimulants. All patients were asked to complete a questionnaire to assess depression, the self‐reported BDI‐II form, and the PSQI to assess sleep quality in the previous weeks. They also completed the ESS to assess daily sleepiness. All patients underwent PSG. After the night of PSG, all participants completed a self‐rated questionnaire on sleep during the night. Actigraphy was performed before or after PSG.

### 2.7 Measurement Forms

#### 2.7.1 Beck Depression Inventory‐II

The self‐reported BDI‐II is a revised version of the depression inventory developed by Aaron Beck and consists of 21 items, with a maximum total score of 63 (Beck et al. 1961). A BDI‐II score between 14 and 19 indicates mild depression; therefore, a cutoff score of 14 was used in this study (Kühner et al. 2007).

#### 2.7.2 Pittsburgh Sleep Quality Index

The PSQI is a self‐reported questionnaire that assesses sleep quality and disturbances within the last month. It comprises seven scores: subjective sleep quality, sleep latency, sleep duration, habitual sleep efficiency, sleep disturbances, use of sleeping medications, and daytime dysfunction. The global score ranges from 0 to 21 points, with a higher score indicating a worse quality of sleep.

#### 2.7.3 Epworth Sleepiness Scale

The ESS is a self‐rated questionnaire that assesses daytime sleepiness. It consists of eight questions on different everyday situations, and the patient rates their likelihood of falling asleep during each situation on a scale of 0–3, where 3 is very likely. Total scores range from 0 to 24, and scores above 10 indicate excessive daytime sleepiness (Gonçalves et al. 2023).

#### 2.7.4 Polysomnography

PSG, the gold standard for objective sleep assessment, includes electroencephalography, electrooculography, chin and limb electromyography, electrocardiography, oxygen saturation monitoring, airflow measurement, and respiratory effort monitoring (Jafari and Mohsenin 2010). To score sleep stages and associated events, the AASM Manual for the Scoring of Sleep and Associated Events (Berry et al. 2017), since 2023 the current version 3, has been used. Both stationary (*n* = 128) and portable (*n* = 96) PSG systems were used (see Section 2.8). In five subjects, the type of PSG (stationary or portable) was not documented. The starting point of the PSG was the observed or self‐documented (portable PSG) time of “lights out” with an intention to go to sleep. Sleep (onset) latency was defined as the duration from lights out to the first 30‐s interval scored as sleep, mostly as the N1 stage.

#### 2.7.5 Self‐Rated PSG Questionnaire

This questionnaire was administered to patients to assess their sleep on the night of PSG. It included questions about sleep duration, sleep latency, wake‐up episodes, awake time after sleep onset, sleep feeling, and dreams (Appendix 2).

#### 2.7.6 Actimetry/Actigraphy

Actigraphy involves the acquisition of data using a movement sensor worn continuously on the nondominant wrist, typically for a week or more (Walia and Mehra 2019). The mean of seven consecutive days/nights was calculated. The SOMNOWatch (SOMNOmedics, Germany) was used in the sleep laboratory.

Four coefficients were used to better assess the subjective–objective mismatch. The sleep‐duration coefficient was calculated by dividing the sleep time subjectively indicated in the self‐rated PSG questionnaire by the sleep time objectively measured by PSG (Coeff_ST_sub_PSG). In contrast to PSG, both actimetry and the PSQI aim to assess sleep over longer periods than one or two nights. We therefore assumed that calculating an index (coefficient) of these two measurements would provide an approximate assumption of mid‐ to long‐term indicators of subjective–objective mismatch. Accordingly, self‐reported TST in PSQI (in minutes) was divided by TST estimated via actimetry (Coeff_ST_PSQI_ACTI). We acknowledge potential limitations, such as differences in the assessment periods (PSQI still covers a longer interval than actimetry) and the indirect nature of measuring sleep duration in actimetry.

The sleep‐latency coefficients were computed using the same approach (Coeff_SL_sub_PSG and Coeff_SL_PSQI_ACTI):

$$\mathrm{Coeff}\_\mathrm{ST}\_\mathrm{sub}\_\mathrm{PSG}=\frac{\mathrm{sleep}\;\mathrm{time}\;\mathrm{in}\;\mathrm{self}\;\mathrm{rated}\;\mathrm{PSG}}{\mathrm{sleep}\;\mathrm{time}\;\mathrm{in}\;\mathrm{PSG}}\;$$

$$\mathrm{Coeff}\_\mathrm{ST}\_\mathrm{PSQI}\_\mathrm{ACTI}=\frac{\mathrm{sleep}\;\mathrm{time}\;\mathrm{in}\;\mathrm{PSQI}}{\mathrm{sleep}\;\mathrm{time}\;\mathrm{in}\;\mathrm{actimetry}}$$

$$\mathrm{Coeff}\_\mathrm{SL}\_\mathrm{sub}\_\mathrm{PSG}=\frac{\mathrm{sleep}\;\mathrm{latency}\;\mathrm{in}\;\mathrm{self}\;\mathrm{rated}\;\mathrm{PSG}}{\mathrm{sleep}\;\mathrm{latency}\;\mathrm{in}\;\mathrm{PSG}}\;$$

$$\mathrm{Coeff}\_\mathrm{SL}\_\mathrm{PSQI}\_\mathrm{ACTI}=\frac{\mathrm{sleep}\;\mathrm{latency}\;\mathrm{in}\;\mathrm{PSQI}}{\mathrm{sleep}\;\mathrm{latency}\;\mathrm{in}\;\mathrm{actimetry}}$$

### 2.8 Statistical Analysis

Descriptive analyses were conducted first to summarize the study sample. Group analysis comparisons between patients with and without comorbid depression were then performed using the nonparametric Wilcoxon rank sum test, based on clinical history or BDI total score (≥14 vs. <14 points), respectively. Finally, as the main component of our analysis, both group comparisons were extended using multivariate linear regression to systematically assess the association between depression comorbidity and individual primary and secondary endpoints, while adjusting for clinically known influential factors. Endpoint (outcome) variables were log‐transformed to account for the positivity and skewness of the vast majority of sleep‐related parameters, such as the nonnegative ranges of the primary endpoints (i.e., subjective–objective coefficients) and the secondary outcomes of standard sleep parameters (e.g., sleep‐stage durations) and clinical scales (e.g., ESS score).

For each endpoint, regression analysis was performed to quantify the effect of depression (defined either as a binary indicator variable based on existing diagnosis or as a BDI score ≥14) while adjusting for a predefined set of demographic and clinical confounders: age, sex, psychopharmacotherapy; use of antidepressants, antipsychotics, or hypnotics; and the presence of OSA syndrome, PLMD, or RLS, all of which are known to interact with insomnia and influence sleep macrostructure (Aalbers et al. 2017; Bonakis et al. 2020; Doghramji and Jangro 2016; Merrill et al. 2023; Peng et al. 2021). Regression analysis also controlled for PSG type (1 = portable, 0 = stationary) to account for potential systematic differences between home‐based and clinical settings. Data from five patients lacking information on PSG type were excluded. Adjustments for medications (e.g., antidepressants) and relevant comorbidities (e.g., OSA) were made in the same manner using binary indicator variables (1 = present; 0 = absent).

Thus, our regression analysis effectively captured the systematic effects of insomnia with and without depression while adjusting for the impact of a broad range of clinically relevant confounders. Despite correlations among some of the covariates (e.g., psychopharmacotherapy and medication classes), all were retained to appropriately disentangle the effects of depression from those of medication. Importantly, we used a fixed set of predictors rather than relying on heuristic variable selection techniques to preserve clinical interpretability and maintain consistency across the models. Statistical analyses were conducted using the statistical program R (version 4.4.3), with a significance level of 0.05, for hypothesis testing.

## 3 Results

We first summarized the patients’ demographic and clinical characteristics using descriptive statistics. We then analyzed the influence of depression on objective and subjective sleep measures using a group analysis as well as multivariate linear regression while controlling for the predefined demographic and clinical confounders. Finally, we evaluated the effects of comorbid depression, defined either as an existing diagnosis or a BDI‐score ≥14, using multivariate regression while controlling for the same set of demographic and clinical confounders.

### 3.1 Descriptive Statistics

**FIGURE 2:**
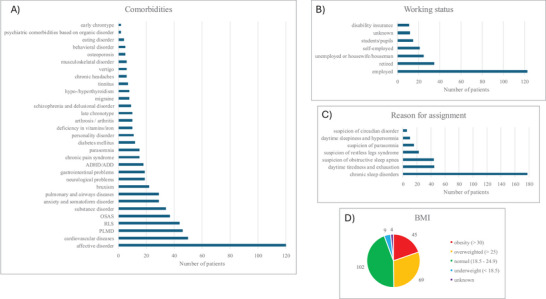
Data for all patients evaluated: (A) comorbidities; (B) working status; (C) reason for assignment; and (D) body mass index. BMI, body mass index; PLMD, periodic limb movement disorder; RLS, restless legs syndrome.

The most common reasons for referral to the sleep laboratory were chronic sleep disturbances (178 patients), daytime tiredness and exhaustion (45 patients), and OSA (44 patients). The mean age of the patients was 47.6 years, and the most common comorbidities were affective disorders (120 patients), cardiovascular disorders (50 patients), PLMD (46 patients), RLS (44 patients), and OSA syndrome (37 patients). More details and the body mass index distribution and working status of the participants are shown in Figure 2.

Among all included patients, 140 (61%) were taking psychotropic medication; 122 (53%) were diagnosed with depression, and 135 (59%) had a BDI score ≥14, indicating mild depression (Table 1).

**TABLE 1:** Population characteristics: Mean, standard deviation, median, and IQR of all variables assessed in 229 subjects.

Variable	Mean	SD	Median	Q25	Q75	IQR
**Age (year)**	47.6	15.11	48	36	57	21
**BMI (kg/m^2^)**	26.02	5.34	25.1	22.2	28.5	6.3
**BDI score (pts)**	18.88	11.38	17	10	26	16
**ESS score (pts)**	6.45	4.41	6	3	9	6
**PSQI total (pts)**	12.39	3.79	13	10	15	5
**PSQI sleep latency (min)**	54.48	51.9	2	1	3	2
**PSQI sleep duration (min)**	346.02	102.13	2	1	3	2
**Self‐rated PSG sleep duration (min)**	380.29	115.9	390	300	450	150
**Self‐rated PSG sleep latency (min)**	49.34	48.39	30	15	60	45
**PSG TST (min)**	367.45	92.72	378	320.5	429	108.5
**PSG sleep efficiency (%)**	76.11	16.09	80.8	68.75	87.85	19.1
**PSG sleep onset latency (min)**	28.41	31.74	17	10	35.5	25.5
**PSG WASO (min)**	86.1	71.59	65	38	110	72
**PSG wake episodes**	19.72	8.73	18	14	25	11
**PSG REM latency (min)**	126.94	80.14	99	68.5	165	96.5
**PSG REM total (min)**	75.94	35.49	76	55	98	43
**PSG N3 total (min)**	21.22	27.54	13	0	31.5	31.5
**PSG mean O_2_ (%)**	94.82	2.09	95	94	96	2
**PSG mean heart rate (bpm)**	63.93	9.08	63	57	69	12
**PSG AHI (per minute)**	2.55	4.64	0.8	0.3	2.18	1.88
**PSG limb arousal index (per hour)**	8.46	9.86	6.5	3.8	9.9	6.1
**ACTI mean sleep time (min)**	398.84	80.96	400	353.5	441.25	87.75
**ACTI mean sleep efficiency (%)**	78.03	9.43	81	74.5	84.3	9.8
**ACTI mean sleep onset latency (min)**	14.63	11.77	12	7	18	11
**ACTI mean WASO (min)**	108.68	99.04	89	66	124.5	58.5
**Coeff_ST_sub_PSG**	1.04	0.32	1.02	0.86	1.17	0.31
**Coeff_SL_sub_PSG**	2.97	3.89	1.69	0.96	3.75	2.79
**Coeff_ST_PSQI_ACTI**	0.92	0.28	0.93	0.75	1	0.25
**Coeff_SL_PSQI_ACTI**	5.36	8.89	2.71	1	6.32	5.32
**Depression (patients)**	53%					
**BDI cut off 14 points (patients)**	59%					
**Psychotropic drugs (patients)**	61%					
**Antidepressants (patients)**	46%					
**Antipsychotics (patients)**	14%					
**Hypnotics (patients)**	25%					
**OSAS (patients)**	16%					
**PLMD (patients)**	20%					
**RLS (patients)**	18%					

Abbreviations: ACTI mean sleep efficiency [%] = mean sleep efficiency in actimetry; ACTI mean sleep onset latency [min] = mean sleep onset latency in actimetry; ACTI mean sleep time [min] = mean sleep duration in actimetry; ACTI mean WASO [min] = mean time awake after sleep onset in actimetry; Age [y] = age in years; Antidepressants [% of patients] = intake of antidepressants; Antipsychotics [% of patients] = intake of antipsychotics; BDI cut off 14 points [patients] = patients with BDI total score of ≥14 points; BDI score [pts] = Beck Depression Inventory; BMI [kg/m^2^] = body mass index; Coeff_SL_PSQI_ACTI = subjective sleep onset latency in PSQI divided by sleep onset latency in actimetry; Coeff_SL_sub_PSG = subjective sleep onset latency in self‐rated PSG form divided by objective sleep onset latency in PSG; Coeff_ST_PSQI_ACTI = subjective sleep duration in PSQI divided by sleep duration in actimetry; Coeff_ST_sub_PSG = subjective sleep duration in self‐rated PSG form divided by TST in PSG; Depression [patients] = patients with diagnosis of depression; ESS score [pts] = epworth sleepiness scale in points; Hypnotics [% of patients] = intake of hypnotics/tranquilizers (benzodiazepines, z‐substances); IQR = interquartile range; OSAS [% of patients] = obstructive sleep apnea syndrome; PLMD [% of patients] = periodic limb movement disorder; PSG AHI [per minute] = Apnea–Hypopnea Index per minute in PSG; PSG limb arousal index [per hour] = Limb arousal index in PSG; PSG mean heart rate [bpm] = mean heart rate PSG; PSG mean O_2_ [%] = mean oxygen saturation in PSG; PSG N3 total [min] = total deep sleep duration in PSG; PSG REM latency [min] = sleep latency in till REM sleep in PSG; PSG REM total [min] = total REM sleep in PSG; PSG sleep efficiency [%] = sleep efficiency in PSG; PSG sleep onset latency [min] = sleep onset latency until first sleep; PSG TST [min] = total sleep time in PSG; PSG wake episodes = number of the wake up episodes in PSG; PSG WASO [min] = wake time after sleep onset in PSG; PSG = polysomnography; PSQI sleep duration [min] = self‐ reported sleep duration in Pittsburgh Sleep Quality Index; PSQI sleep latency [min] = self‐reported sleep‐onset latency in Pittsburgh Sleep Quality Index; PSQI total [pts] = Pittsburgh Sleep Quality Index; Psychotropic drugs [% of patients] = intake of one or more psychopharmaceuticals; Q25 = 25th percentile; Q75 = 74th percentile; RLS [% of patients] = restless legs syndrome; SD = standard deviation; Self‐rated PSG sleep duration [min] = sleep duration in self‐rated PSG form; Self‐rated PSG sleep latency [min] = sleep latency in self‐rated PSG form.

Patients slept an average of approximately 6 h in the PSG and approximately 30 min longer, according to actimetry. The mean sleep latency varied from approximately 15 min to just under 1 h across the different sleep measures (54 min in the PSQI, 49 min in the self‐rated PSG form, 29 min in PSG, and 15 min in actimetry). On average, patients were awake between 1.5 and 2 h during the night. Overall, the cohort slightly underestimated their sleep duration in long‐term measurements but slightly overestimated it in short‐term measurements. The mean Coeff‐ST‐PSQI_ACTI was 0.92 (one sample *t*‐test: *t* = −2.5516, df = 69, *p* = 0.01294, 95% CI 0.849–0.982), and the mean Coeff_ST_sub_PSG was 1.04 (*t* = 1.8044, df = 165, *p* = 0.07299, 95% CI 0.995–1.095), with values of 1.11 for stationary PSG and 0.95 for portable PSG. In contrast, participants markedly overestimated their sleep latency in both long‐term and short‐term measurements. The mean Coeff_SL_sub_PSG was 2.97 (*t* = 6.6729, df = 173, *p* < 0.001, 95% CI 2.405–3.579), with values of 2.88 for stationary and 3.13 for portable PSG. The mean Coeff_SL_PSQI_ACTI was 5.36, showing a highly significant difference from the null value (coeff. 1.0: *t* = 3.7947, df = 59, *p* = 0.0003508, 95% CI 3.059–7.651).

### 3.2 Group Analysis

To address the primary research goal, two group analyses were performed: one for the differences between patients with insomnia with comorbid depressive disorders and those without and a second one for patients with insomnia with a BDI total score ≥14 (regardless of a history of depression) and those with insomnia and a BDI total score <14. The Wilcoxon rank‐sum test (also known as the Mann–Whitney *U* test) was used (as the majority of the data were not normally distributed). Table 2 summarizes the results of both groups.

**TABLE 2:** Group differences calculated with the Wilcoxon rank sum test.

	Group analysis: depressive disorder vs. without depressive disorder					Group analysis: BDI ≥14 points vs. BDI <14 points				
Variable	Mean in group without depressive disorder	Mean in group with depressive disorder	W	*p* value	Effect size	Mean in group with total BDI <14 pts	Mean in group with total BDI ≥14 pts	W	*p* value	Effect size
**BDI score (pts)**	14.36	22.95	3209	<0.001	0.386	7.89	25.36	0	<0.001	0.837
**ESS score (pts)**	5.97	6.84	4872.5	0.132	0.104	6.61	6.36	5246	0.794	0.018
**PSQI total (pts)**	12.59	12.18	4820	0.509	0.048	11.45	13.03	3336	0.011	0.184
**PSQI sleep quality (pts)**	2.31	2.06	5736	0.026	0.158	1.95	2.31	3300.5	<0.001	0.250
**PSQI sleep latency (pts)**	1.98	2.05	4576.5	0.71	0.027	1.76	2.2	3447.5	0.0062	0.197
**PSQI sleep duration (pts)**	1.87	1.48	5417	0.016	0.174	1.72	1.64	4409	0.58	0.041
**PSQI sleep efficiency (pts)**	1.79	1.66	4629.5	0.5	0.048	1.63	1.81	3775.5	0.36	0.067
**PSQI sleep disturbances (pts)**	1.46	1.59	4380.5	0.12	0.110	1.34	1.65	3393	<0.001	0.247
**PSQI medication (pts)**	1.49	1.65	4458.5	0.4	0.061	1.45	1.68	4070	0.27	0.080
**PSQI daytime disturbance (pts)**	1.67	1.94	4109.5	0.019	0.165	1.58	1.98	3446	<0.001	0.234
**PSQI sleep latency (min)**	52.33	55.66	4576.5	0.706	0.027	42.94	61.54	3447.5	0.00621	0.197
**PSQI sleep duration (min)**	326.76	363.2	5417	0.0162	0.174	339.17	350.13	4409	0.577	0.041
**Self‐rated PSG sleep duration (min)**	381.58	378.2	4109.5	0.802	0.019	369.81	381.67	3301	0.436	0.059
**Self‐rated PSG sleep latency (min)**	44.06	53.51	3783	0.0381	0.149	40.25	55.05	3013.5	0.0197	0.173
**PSG TST (min)**	381.17	356.41	7125.5	0.0835	0.115	358.25	371.17	4771	0.162	0.096
**PSG sleep efficiency (%)**	79.27	73.39	7493.5	0.0129	0.166	76.65	75.6	5244.5	0.747	0.022
**PSG sleep onset latency (min)**	23.05	33.07	5032	0.0101	0.172	26.16	29.86	5195	0.664	0.030
**PSG WASO (min)**	74.3	96.7	5414	0.0742	0.119	78.74	91.02	5361	0.955	0.004
**PSG wake episodes**	19.48	20.06	6148.5	0.783	0.018	18.72	19.91	4913	0.281	0.074
**PSG REM latency (min)**	111.2	140.94	4786	0.0202	0.158	114.1	135.76	4302.5	0.0863	0.120
**PSG REM total (min)**	80.75	72.07	7213	0.056	0.127	76	75.41	5425.5	0.93	0.006
**PSG N3 total (min)**	22.82	19.83	6828.5	0.258	0.075	24.64	19.23	5611.5	0.606	0.035
**PSG mean O_2_ (%)**	95.08	94.6	5314	0.35	0.066	94.96	94.75	4370	0.614	0.037
**PSG mean heart rate (bpm)**	63.28	64.49	5439.5	0.321	0.067	62.76	64.55	4450.5	0.14	0.102
**PSG AHI (per minute)**	2.19	2.9	5862.5	0.445	0.051	3.19	2.27	5609	0.547	0.041
**PSG limb arousal index (per hour)**	9.82	7.35	6719.5	0.245	0.078	10.12	7.58	5781.5	0.247	0.080
**ACTI mean sleep time (min)**	375.87	414.67	474	0.0184	0.271	385.21	408.79	543.5	0.212	0.145
**ACTI mean sleep efficiency (%)**	76.87	78.85	555.5	0.175	0.157	77.21	78.88	584.5	0.578	0.065
**ACTI mean sleep onset latency (min)**	15.25	14.21	734	0.703	0.044	14.78	14.45	716	0.528	0.073
**ACTI mean WASO (min)**	97.06	116.87	674	0.936	0.010	98.22	112.94	613	0.814	0.028
**Antidepressants (number of participants)**	0.26	0.66	3772.5	<0.001	0.400	0.308	0.548	4092.5	<0.001	0.233
**Depression (number of participants)**	0	1	NA	NA	NA	0.3625	0.629	3957.5	<0.001	0.258
**Coeff_ST_sub_PSG**	0.99	1.09	2958.5	0.0628	0.143	0.999	1.06	2703	0.318	0.079
**Coeff_SL_sub_PSG**	3.02	2.95	4082	0.515	0.049	2.45	3.32	2750	0.143	0.113
**Coeff_ST_PSQI_ACTI**	0.91	0.92	596.5	0.851	0.023	0.95	0.89	633	0.529	0.076
**Coeff_SL_PSQI_ACTI**	3.53	6.41	330	0.179	0.174	3.85	6.29	335	0.222	0.160

*Note*: Significant differences are in orange. **PSQI sleep quality [pts]** = score on the corresponding PSQI‐item; **PSQI sleep latency [pts]** = score on the corresponding PSQI‐item; **PSQI sleep duration [pts]** = score on the corresponding PSQI‐item; **PSQI sleep efficiency [pts]** = score on the corresponding PSQI‐item; **PSQI sleep disturbances [pts]** = score on the corresponding PSQI‐item; **PSQI medication [pts]** = score on the corresponding PSQI‐item; **PSQI daytime disturbance [pts]** = score on the corresponding PSQI‐item. Effect size categories for the Wilcoxon rank‐sum test: 0.10–<0.3 = small effect; 0.30–<0.5 = moderate effect; ≥0.5 = large effect. For all other variables, see Table 1 footnote.

Abbreviation: NS = not significant.

Patients with insomnia diagnosed with a depressive disorder showed significantly higher scores on the PSQI daytime disturbances item but significantly lower scores on the PSQI sleep‐duration and sleep‐quality items (with lower scores indicating better subjective sleep duration and quality). Both self‐rated and PSG‐measured sleep‐onset latencies were significantly longer, whereas sleep efficiency was significantly lower, than in patients with insomnia without comorbid depression. Similar to the PSQI, TST estimated by actigraphy was significantly higher.

In the group analysis based on symptom severity defined by the BDI total score cutoff, patients with insomnia with a BDI total score of ≥14 showed a significantly higher PSQI total score as well as higher scores in PSQI items sleep quality, sleep latency, sleep latency in minutes, sleep disturbances, and daytime disturbances.

Next, we conducted linear regression analyses with log‐transformed outcomes to control for age, sex, psychopharmacotherapy, and intake of antidepressants, antipsychotics, or hypnotics, as well as OSA syndrome, PLMD, and RLS. PSG type (stationary or portable) was included as a predictor. Log‐linear regression results for depression as a trait (based on history) and as a state (BDI ≥14) are summarized in Table 3.

**TABLE 3:** Group differences assessed using linear regression with log‐transformed outcomes in 224 subjects.

**Outcome**	**Mean in group without depression**	**Mean in group with depression**	*W*	*p* value	Intercept	**Depression**	Age >50 year	Sex F	Antidepressants	Psychopharmaceutics	Antipsychotics	Hypnotics	OSAS	PLMD	RLS	Portable PSG
**BDI score (pts)**	14.36	22.95	3209	<0.001	10.57 (***)	1.7 (***)	0.88 (**)	0.95 (NS)	1.72 (**)	0.63 (*)	1.29 (NS)	1.18 (NS)	1.07 (NS)	0.97 (NS)	0.96 (NS)	
**PSQI sleep quality (pts)**	2.31	2.06	5736	0.026	2.17 (***)	0.85 (NS)	1.05 (NS)	1.08 (NS)	0.98 (NS)	0.87 (NS)	1.12 (NS)	1.25 (NS)	0.76 (NS)	1.15 (NS)	1.02 (NS)	
**PSQI sleep duration (pts)**	1.87	1.48	5417	0.016	1.33 (NS)	0.62 (NS)	1.76 (***)	1.47 (NS)	0.84 (NS)	0.79 (NS)	0.92 (NS)	0.62 (NS)	1 (NS)	0.76 (NS)	0.91 (NS)	
**PSQI daytime disorder (pts)**	1.67	1.94	4109.5	0.019	1.03 (NS)	1.1 (NS)	0.83 (**)	0.84 (NS)	1.43 (NS)	1.11 (NS)	0.84 (NS)	1.17 (NS)	1.13 (NS)	0.95 (NS)	1.21 (NS)	
**PSQI sleep duration (min)**	326.76	363.2	3614	0.016	309.26 (***)	1.11 (*)	0.93 (***)	0.94 (NS)	1.08 (NS)	0.93 (NS)	1.05 (NS)	1.1 (NS)	0.97 (NS)	1.04 (NS)	0.98 (NS)	
**Self‐rated PSG sleep latency (min)**	44.06	53.51	3783	0.038	30.3 (***)	1.21 (NS)	0.96 (NS)	1.04 (NS)	0.95 (NS)	1.14 (NS)	0.84 (NS)	1.25 (NS)	1.17 (NS)	1.06 (NS)	1.15 (NS)	0.58 (***)
**PSG TST (min)**	381.17	356.41	7125.5	0.083	304.04 (***)	0.89 (*)	0.93 (***)	1.18 (***)	0.98 (NS)	1.04 (NS)	1.08 (NS)	1.04 (NS)	1.07 (NS)	0.98 (NS)	1.15 (NS)	1.09 (NS)
**PSG sleep efficiency (%)**	79.27	73.39	7493.5	0.013	66.91 (***)	0.89 (*)	0.94 (***)	1.11 (*)	1.03 (NS)	0.98 (NS)	1.05 (NS)	1.03 (NS)	1.04 (NS)	1.02 (NS)	1.09 (NS)	1.13 (**)
**PSG sleep onset latency (min)**	23.05	33.07	5032	0.01	17.26 (***)	1.41 (*)	0.98 (NS)	0.97 (NS)	0.92 (NS)	1.16 (NS)	0.96 (NS)	1.06 (NS)	0.83 (NS)	1.29 (NS)	0.83 (NS)	0.63 (***)
**PSG REM latency (min)**	111.2	140.94	4786	0.02	90.18 (***)	1.09 (NS)	1 (NS)	1.04 (NS)	1.24 (NS)	1.12 (NS)	0.99 (NS)	1 (NS)	1.11 (NS)	0.94 (NS)	0.99 (NS)	0.83 (*)
**PSG limb arousal index (per hour)**	9.82	7.35	6719.5	0.24	6.09 (***)	0.69 (**)	1.01 (NS)	0.84 (NS)	1.26 (NS)	1.03 (NS)	0.87 (NS)	0.89 (NS)	1.12 (NS)	1.77 (***)	1.53 (*)	0.9 (NS)
**ACTI mean sleep time (min)**	375.87	414.67	474	0.018	356.71 (***)	1.14 (*)	0.99 (NS)	1.04 (NS)	0.75 (***)	1.32 (**)	0.91 (NS)	0.92 (NS)	0.88 (NS)	0.95 (NS)	1.06 (NS)	
	**Mean in group with BDI <14 pts**	**Mean in group with BDI ≥14 pts**	*W*	*p* value	Intercept	**BDI cutoff**	Age >50 year	Sex F	Antidepressants	Psychopharmaceutics	Antipsychotics	Hypnotics	OSAS	PLMD	RLS	Portable PSG
**BDI score (pts)**	7.89	25.36	0	<0.001	6.45 (***)	3.47 (***)	0.93 (NS)	1 (NS)	1.33 (*)	0.84 (NS)	1.02 (NS)	0.97 (NS)	1.11 (NS)	1.03 (NS)	0.88 (NS)	
**PSQI total (pts)**	11.45	13.03	3336	0.011	10.12 (***)	1.22 (***)	1.07 (**)	1.1 (NS)	0.99 (NS)	1.01 (NS)	1.01 (NS)	1.09 (NS)	0.9 (NS)	1.06 (NS)	0.98 (NS)	
**PSQI sleep quality (pts)**	1.95	2.31	3300.5	<0.001	1.88 (***)	1.36 (**)	1.1 (*)	1.05 (NS)	0.88 (NS)	0.81 (NS)	1.16 (NS)	1.26 (NS)	0.74 (*)	1.15 (NS)	0.94 (NS)	
**PSQI sleep latency (pts)**	1.76	2.2	3447.5	0.0062	0.56 (NS)	1.98 (*)	0.99 (NS)	0.91 (NS)	0.83 (NS)	1.51 (NS)	1.39 (NS)	1.05 (NS)	1.16 (NS)	1.62 (NS)	1.17 (NS)	
**PSQI sleep efficiency (pts)**	1.63	1.81	3775.5	0.36	0.25 (***)	2.38 (*)	1.68 (***)	3.5 (***)	0.63 (NS)	1.33 (NS)	0.93 (NS)	0.81 (NS)	0.91 (NS)	1.18 (NS)	1.13 (NS)	
**PSQI sleep disturbances (pts)**	1.34	1.65	3393	<0.001	1.18 (NS)	1.48 (***)	1.1 (**)	1.27 (**)	1.01 (NS)	0.89 (NS)	0.84 (NS)	0.9 (NS)	0.95 (NS)	0.98 (NS)	0.84 (NS)	
**PSQI daytime disorder (pts)**	1.58	1.98	3446	<0.001	0.94 (NS)	1.25 (NS)	0.84 (**)	0.86 (NS)	1.37 (NS)	1.16 (NS)	0.8 (NS)	1.14 (NS)	1.15 (NS)	0.96 (NS)	1.19 (NS)	
**PSQI sleep latency (min)**	42.94	61.54	3038	<0.001	23.09 (***)	1.62 (*)	1.01 (NS)	1.08 (NS)	0.69 (NS)	1.76 (NS)	0.69 (NS)	0.83 (NS)	0.86 (NS)	1.14 (NS)	0.79 (NS)	
**Self‐rated PSG sleep latency (min)**	40.25	55.05	3013.5	0.0197	27.15 (***)	1.45 (*)	0.97 (NS)	1.03 (NS)	0.83 (NS)	1.39 (NS)	0.72 (NS)	1.15 (NS)	1.17 (NS)	1.01 (NS)	1.17 (NS)	0.56 (***)

*Note*: The effects are presented as exp(β) to facilitate interpretation. For example, an exp‐effect of 1.7 for depression on BDI score corresponds to a 70% higher BDI in patients with insomnia and comorbid depression compared to those without depression. *mild significance (0.01 ≤ *p* < 0.05); **strong significance (0.001 ≤ *p* < 0.01); ***very strong significance (*p* < 0.001). For all other variables, see Tables 1 and 2.

Abbreviation: NS = not significant.

When controlling for influencing factors, significant group differences emerged regarding the diagnosis of depressive disorder. PSQI sleep duration in minutes, PSG sleep‐onset latency, and actigraphy‐derived TST were significantly higher, whereas PSG‐measured TST and sleep efficiency were significantly lower in patients with insomnia with comorbid depression than in those without depression. For example, a coefficient of 1.4 for PSG sleep‐onset latency in patients with insomnia with depression compared to insomnia without depression refers to an increase of 40%.

In patients with insomnia with a BDI total score of ≥14, the following factors were significantly higher than in patients with insomnia with a BDI score of 0–13: PSQI total score, PSQI sleep quality, PSQI sleep latency as a score and in minutes, PSQI sleep disturbances, and the self‐rated PSG sleep latency.

**FIGURE 3:**
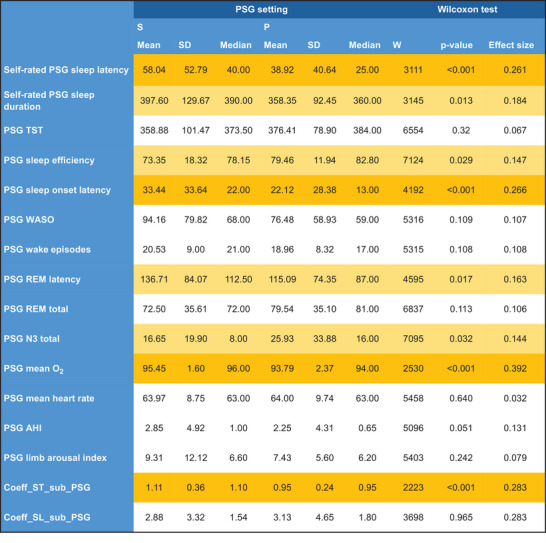
Summary of all polysomnography (PSG)‐related measures and comparison of stationary and portable PSG. REM, rapid eye movement sleep; TST, total sleep time.

When stationary and portable PSG recordings were analyzed separately, significant group differences were found for the subjective parameters self‐rated sleep‐onset latency, perceived TST, and the subjective/objective TST coefficient as well as the objective measures sleep‐onset latency, sleep efficacy, REM latency, N3 (non‐rapid eye movement sleep stage 3, the deep/slow wave sleep stage) sleep duration, and mean O_2_ saturation (Figure 3).

## 4 Discussion

The main findings of our study are that there are different profiles of sleep disturbances in patients with insomnia, depending on whether they have comorbid depressive disorders or manifest depressive symptoms. Although the diagnosis of depression (as a trait) was associated with significant changes in objective measures (longer sleep‐onset latency and lower sleep efficiency in PSG as well as longer sleep duration in actimetry), patients classified by BDI score ≥14 points showed significant impairment in subjective sleep measures only (PSQI). One possible explanation could be that the subjective–objective mismatch is primarily related to the current severity of (in this case, depressive) psychopathology, whereas the objective changes in sleep related to depression are more substantial and, to some extent, independent of symptom severity. This is supported by the literature showing longer sleep‐onset latency and lower sleep efficiency in subjects with depressive disorders (Augustinavicius et al. 2014; Omichi et al. 2022).

Both the subjective total sleep duration reported in the PSQI and the midterm sleep duration estimated from actimetry were significantly longer in participants diagnosed with depression. This phenomenon is currently a topic of debate in the literature, as patients with depression show both shorter and longer sleep duration (Difrancesco et al. 2019) and both short and long TST values are associated with a higher risk of depression (Chunnan et al. 2022; Zhai et al. 2015). Notably, a longer actimetry‐derived TST is an approximation based on measurements of activity. Because a sedentary lifestyle and longer time spent in bed are associated with depressive symptoms (Achttien et al. 2019; Xu et al. 2024), we hypothesized that actimetry may overestimate TST in patients with major depressive disorder.

Patients with insomnia and high BDI scores did not differ from those with low BDI scores in objective sleep measures; however, they reported greater subjectively perceived daytime impairment and more sleep disturbances. These results confirm previous findings where the subjective–objective mismatch in sleep parameters was related to the severity of depressive symptoms (Tsuchiyama et al. 2003).

These findings highlight the strong influence of current depressive symptomatology on subjective sleep perceptions and are consistent with previous research showing that acute depressive symptoms intensify negative sleep appraisals independent of objective sleep measures (Harvey 2002). Interestingly, regression analysis revealed that female sex was an independent predictor of higher scores on the PSQI sleep efficiency item among patients with higher BDI total scores, which warrants further investigation.

Furthermore, all patients with insomnia (with and without comorbid depression) estimated their subjective sleep duration with high accuracy, according to the morning protocol and the PSQI when compared with PSG and actimetry (subjective/objective coefficients of 1.04 for the PSG night and 0.92 for actimetry). A more accurate perception of TST for the PSG night than for actimetry aligns with the findings of Maltezos et al. (2024), although the difference was marginal. The slight overestimation of sleep duration by the majority of participants (mean coefficient 1.04) contrasts with common findings in patients with insomnia, who typically tend to underestimate their TST.

Possible reasons for this finding are discussed below. The substantial number of patients diagnosed with depression (mean BDI total score >18) in our sample could be one possible explanation for the lack of TST underestimation, which is typical in patients with insomnia. Indeed, patients with depression demonstrate both underestimation and overestimation of TST (Difrancesco et al. 2019; Tsuchiyama et al. 2003).

The subjective–objective sleep mismatch in insomnia shows greater variability across studies than in good sleepers, ranging from an underestimation of TST by 3 h to an overestimation of 10 min (Stephan and Siclari 2023). As the TST overestimation of 4% in our study was not significantly increased when compared to a coefficient of 1.0 (no subjective–objective mismatch), the results can be seen as within the normal range. Furthermore, the comparison of stationary and portable measures revealed that TST was overestimated only in the stationary PSG.

Sleep duration and quality are affected by psychological and physiological conditions, culture, and environmental factors (Troynikov et al. 2018). The overestimation of TST under laboratory conditions could be attributed to conditioning in a home setting for insomnia (one's own bedroom is associated with insomnia rather than sleep). Independent of the well‐described first‐night effect in sleep laboratories in patients with insomnia (Hu, Shi, et al. 2024), patients with primary insomnia reported increased subjective sleep efficacy on two consecutive laboratory nights compared to home settings (Hirscher et al. 2015). Finally, the effect of medication on the subjective–objective mismatch is poorly understood.

In contrast, in the midterm assessment, a general underestimation of TST (PSQI/actimetry, coefficient = 0.92, *p* = 0.013 compared to the null value) was observed.

Consistent with previous findings, stationary PSG was associated with a longer REM latency (Hu, Chen, et al. 2024). The authors note that future work will focus on the associations between confounding factors and sleep parameters within the present sample.

Sleep latency was overestimated by factors of 3 and 5 in both the stationary and portable PSG. This significant overestimation is consistent with previous findings on sleep latency estimation in patients with insomnia (Harvey and Tang 2012; Smith and Trinder 2000; Valko et al. 2021). Some findings indicate that patients with insomnia overestimate sleep latency but are equally likely to underestimate or overestimate TST, depending on comorbid disorders and individual psychopathology (Vanable et al. 2000). We assume that this was the case in our sample, whereby the overestimation of sleep‐onset latency is a common feature of both insomnia and depressive symptoms.

In summary, our study provides new insights into the subjective–objective mismatch in patients with insomnia, comorbid depressive disorders, or depressive symptoms. In particular, the subjective perception of sleep disturbances as well as the perceived daytime symptoms can be strongly affected by relevant depressive symptoms. These findings underscore the importance of assessing depressive symptoms in patients with insomnia. Further investigations are required to gain a better understanding of the affective (depressive) components of insomnia. The combination of subjective and objective measures appears to be beneficial and should be recommended when dealing with clinical samples.

The present study has several limitations. First, the cutoff for depression was based on a self‐rating scale (BDI), and no expert rating scale for depression was administered. Second, the “real‐world” sample, characterized by various comorbidities as well as the use of psychotropic medications, which can also be considered a strength, makes it challenging to draw causal conclusions about the relationship among insomnia, depression, sleep patterns, and potential confounders.

## 5 Conclusions

Our results indicate that in patients with insomnia, the current subjective burden of depressive symptoms (i.e., manifested depressive symptoms) increases the subjective–objective mismatch, as measured by the PSQI, independent of the diagnosis of depression according to the International Classification of Diseases (ICD)‐10 criteria. In patients with insomnia, depression, or depressive symptoms, the subjective assessment of sleep duration can still be accurate, whereas the perceived sleep‐onset latency can be overestimated three to five times.

## Author Contributions

**Tina Carbonetti**: writing – original draft, visualization, writing – review and editing, data curation, investigation. **Michal Bechny**: formal analysis, software, writing – review and editing, validation. **Corrado Garbazza**: methodology, formal analysis, writing – original draft. **Jana Koprivova**: writing – original draft, writing – review and editing. **Karolina Janku**: methodology, writing – review and editing. **Helen Christina Slawik**: writing – review and editing, resources. **Annette Beatrix Bruehl**: project administration, resources. **Undine Emmi Lang**: resources, supervision. **Jan Sarlon**: conceptualization, investigation, methodology, project administration, supervision, writing – review and editing.

## Funding

The authors have nothing to report.

## Disclosure

Coded data can be shared on demand during the review process.

## Conflicts of Interest

The authors declare no conflicts of interest.

## Data Availability

The data that support the findings of this study are available on request from the corresponding author. The data are not publicly available due to privacy or ethical restrictions.
